# Blue light-induced phototoxicity in retinal cells: implications in age-related macular degeneration

**DOI:** 10.3389/fnagi.2024.1509434

**Published:** 2024-12-17

**Authors:** Harshini Chakravarthy, Vasil Georgyev, Cole Wagen, Amir Hosseini, Joanne Matsubara

**Affiliations:** Department of Ophthalmology and Visual Sciences, Faculty of Medicine, University of British Columbia, Vancouver, BC, Canada

**Keywords:** phototoxicity, retinal inflammation, photoreceptors, retinal pigmented epithelial (RPE) cells, age-related macular degeneration (AMD), blue light exposure, LED, blue light

## Abstract

Sunlight exposure is recognized as a risk factor for the development of age-related macular degeneration (AMD), a common neurodegenerative retinal disease in the elderly. Specifically, the blue light wavelengths within sunlight can negatively impact the physiology of light-sensitive retinal cells, including retinal pigmented epithelium (RPE) and photoreceptors. This review explores blue light-induced retinal degeneration, emphasizing the structural and functional impairments in RPE. The initial section provides a brief overview of blue light’s effects on photoreceptors, followed by a comprehensive analysis of its detrimental impact on RPE. *In vitro* studies reveal that blue light exposure induces morphological alterations and functional impairments in RPE, including reduced phagocytic activity, disrupted secretion of neurotrophic factors, and compromised barrier function. Mechanisms of retinal damage, including oxidative stress, inflammation, lipofuscin accumulation, mitochondrial dysfunction and ER stress in RPE, are also explored. The strengths and limitations of *in vitro*, animal and *ex vivo* models for studying blue light exposure are discussed, with recommendations for improving reproducibility in future studies.

## Introduction

Age-related macular degeneration (AMD) is a degenerative retinal disease affecting the macula, leading to progressive central vision loss and is the leading cause of irreversible blindness in people aged 50 and older (Steinmetz et al., 2021). The pathogenesis of AMD is multifactorial, involving environmental and genetic factors. Several early changes are associated with aging, a major risk factor for AMD. Environmental risk factors include sunlight exposure, cigarette smoke and oxidative stress linked to age and diet (Evans, 2005; Jonasson et al., 2014; Lambert et al., 2016; Schick et al., 2016).

Sunlight, specifically the wavelengths associated with ultraviolet (UV) and blue light, impacts the physiology of retinal cells and is, to some extent, comparable to the photo-aging of the skin (Parkinson et al., 2015). An important distinction is that retinal cells may be affected by certain wavelengths of sunlight, such as blue light (400–500 nm), since most of the radiation in the UV range is absorbed by the cornea (100–315 nm) and lens (315–400 nm), thereby helping to protect the retina from UV-induced photo-oxidative damage (Behar-Cohen et al., 2011; Mallet and Rochette, 2011, 2013).

In recent years, there have been growing concerns about the long-term effects of artificial light exposure from light emitting diodes (LEDs) and modern electronic devices which emit a high proportion of their light in the blue wavelength (Behar-Cohen et al., 2011; Wong and Bahmani, 2022). Research in the field indicates that chronic exposure to blue light in the range of 400–490 nm may affect the function of photoreceptor and RPE due to its high energy, and contribute to the pathogenesis of AMD (Roehlecke et al., 2009; Lin et al., 2017; Gea et al., 2018; Baker et al., 2022; Françon et al., 2024). Nevertheless, evidence from clinical studies remains inconclusive and a definitive link between blue light and retinal damage has yet to be established.

## Blue light: a risk factor for AMD?

Estimating the link between sunlight or blue light exposure and AMD risk is challenging due to inconsistent results from population-based studies and meta-analyses of the epidemiological literature (Cruickshanks et al., 2001; Tomany, 2004; Sui et al., 2013; Schick et al., 2016; Zhou et al., 2018; Achiron et al., 2021). The Beaver Dam Eye Study found a possible association between sunlight exposure and increased risk of retinal pigmented epithelium (RPE) abnormalities and early AMD (Cruickshanks et al., 2001; Tomany, 2004). The Chesapeake Bay study reported that AMD patients with extensive geographic atrophy had significantly higher exposure to blue light over the preceding 20 years, compared to age-matched controls (Taylor et al., 1990). The authors combined personal exposure histories with laboratory investigations and field measurements to determine ocular exposure to sunlight. Published ambient data on intensity and spectral distribution of visible light was used to calculate the yearly ocular exposure to blue light for each individual. In the European Eye Study (EUREYE), blue light exposure was estimated by combining meteorologic and questionnaire data regarding outdoor exposure. No link was found between blue light exposure and neovascular or early AMD. However, significant associations were found between blue light exposure and wet AMD in participants with low levels of antioxidants, specifically dietary zinc and zeaxanthin, vitamins C and E in blood. These nutrients are known to work synergistically to protect the retina from light-induced oxidative damage (Thomson et al., 2002; Wrona et al., 2003, 2004). This study emphasizes the complexity of these associations, and the need for further research in this direction (Fletcher, 2008).

Unlike sunlight, artificial lighting contains a fixed spectral distribution that peaks in the blue portion of the electromagnetic spectrum, and the long-term impact of chronic exposure to artificial light on retinal health is unclear (Contín et al., 2016; Wong and Bahmani, 2022). A recent case–control study conducted using nationwide population-based data in South Korea found that artificial light exposure at night significantly increased the risk of developing exudative AMD (Kim et al., 2024). Excessive blue light exposure consistently damages photoreceptors and RPE in culture and animal models, as detailed in the following two sections. However, this correlation has not yet been fully validated in human studies and remains an area of active research.

## Effect of blue light on photoreceptors

Photoreceptors are specialized sensory cells in the retina and interact with the RPE to convert incoming light into neural signals, a process known as phototransduction (Molday and Moritz, 2015). Photoreceptor health is crucial for vision. Light-induced damage is classified into two types based on total dose, which includes irradiance (mW/cm^2^) and exposure duration: Class I, or Noell damage, predominantly studied in rats but also observed in primates and other species, occurs with longer exposures (>1.5 h) and lower irradiance (<1 mW/cm^2^), and primarily affects photoreceptors, although RPE damage can occur with extended exposure (>8 days) (Noell et al., 1966; Noell, 1980; Hunter et al., 2012). Class II, or Ham damage, predominantly studied in primates but also observed in rats and other species, results from shorter exposures (<5 h) at much higher irradiances (>10 mW/cm^2^), and mainly affects the RPE (Ham et al., 1979; Ham, 1983; Hunter et al., 2012; Cougnard-Gregoire et al., 2023; Youssef et al., 2024; Zhang et al., 2024).

In rats, blue light damages photoreceptors through several cellular mechanisms, including reactive oxygen species (ROS) production, mitochondrial damage, and apoptosis (Busch et al., 1999; Theruveethi et al., 2022). In primates, blue light caused significantly more damage to the RPE and cone outer segments compared to longer wavelengths (Zhang et al., 2024). In rats,12 h/day of blue light for 28 days significantly disrupted the outer retinal structure, notably reducing photoreceptor nuclei, damaging their outer segments, and disrupting the outer plexiform layers (Theruveethi et al., 2022). Photoreceptor transcriptome profiling by RNA-seq after blue light exposure in *Drosophila* demonstrated an upregulation of a broad range of genes involved in oxidative stress response and neuroprotective pathways, with concomitant downregulation of genes required for light response including voltage-gated calcium, potassium and chloride ion channels. Interestingly, mature flies were more susceptible to these blue light-induced transcriptomic changes compared to very young flies (Hall et al., 2018).

**Figure 1:**
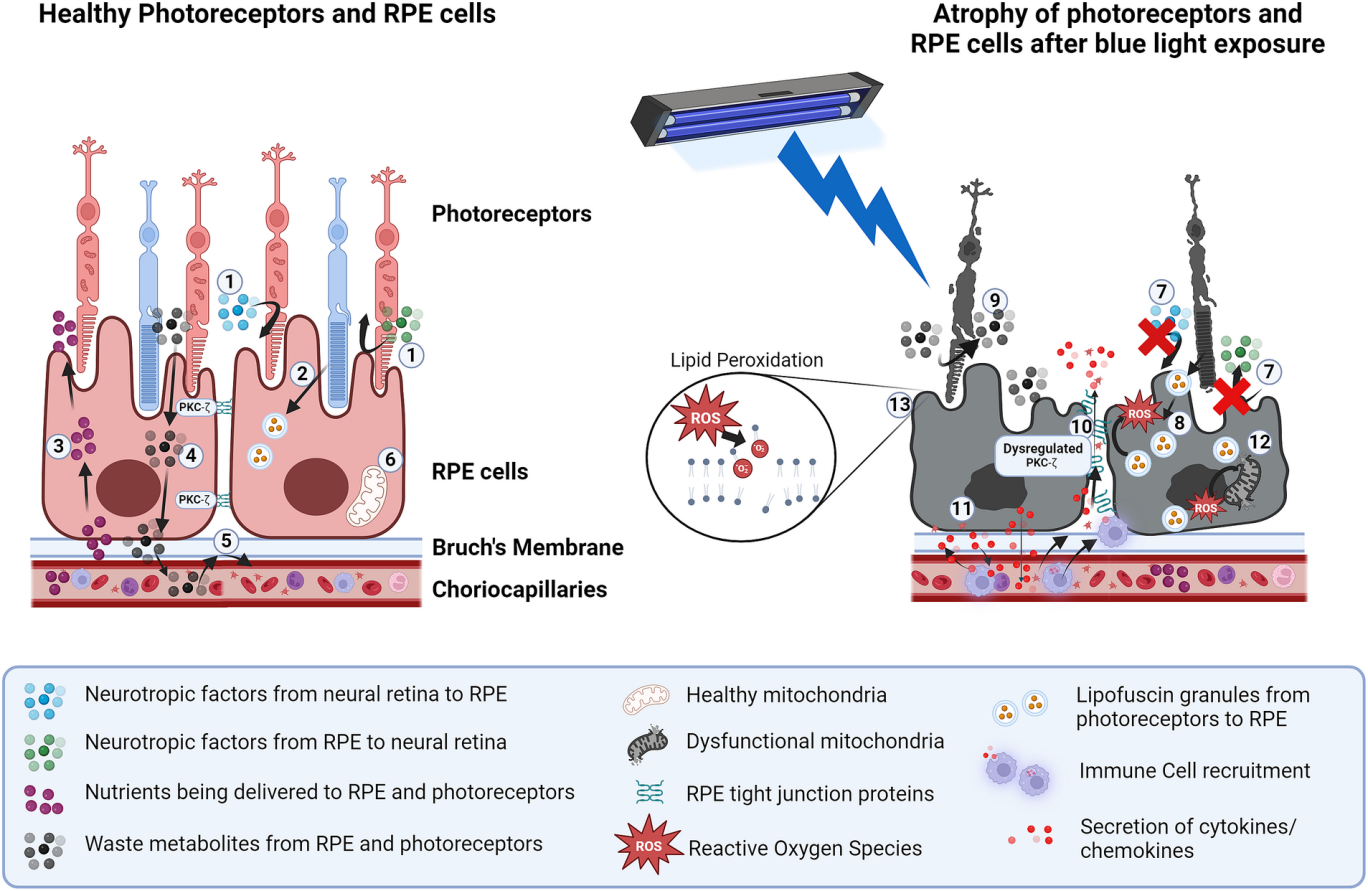
Structural and functional impairments in photoreceptors and RPE cells due to blue light exposure. *In healthy photoreceptors and RPE cells:* 1. secreted neurotropic factors such as HGF, VEGF and angiogenin help maintain homeostasis of the neural retina and RPE. 2. Phagocytosis of photoreceptor outer segments by RPE into lysosomes helps preserve photoreceptor function. 3. Nutrient uptake is controlled by RPE. 4. Metabolite exchange from retina to choroid is controlled by RPE. 5. Outer blood-retinal barrier is maintained due to RPE tight junctions. 6. Healthy mitochondria help manage oxidative stress in RPE. *Blue light exposure of RPE causes:* 7. Dysfunctional neurotrophic factors secretion between neural retina and RPE. 8. Dysfunction in phagocytosis of photoreceptor outer segments 9. Metabolite accumulation in the retina due to lack of uptake by RPE. 10. Breakdown of tight junctions due to blue light-induced dysfunction of PKC-*ζ*. 11. Secretion of cytokines/chemokines causes inflammation and exacerbates the recruitment of immune cells which further damage RPE. 12. Increase in oxidative stress from lipofuscin granules and ROS resulting in mitochondrial damage. 13. Lipid peroxidation from ROS resulting in cellular toxicity and reduced viability. *Created in BioRender.* BioRender.com/e21v624.

In cultured murine photoreceptor-derived cells, blue light increased production of ROS, altered protein expression, and caused photoreceptor damage (Kuse et al., 2014). Photoreceptor cell death from blue light exposure was mainly caused by inducing mitochondrial dysfunction and apoptosis through Bax and caspase-3 activation (Xu et al., 2023). Similarly, mouse retinal explant cultures showed increased ROS production, morphological changes including the disorganization of the outer segments, cell membrane disruption, and cell death with prolonged blue light exposure (Figure 1) (Roehlecke et al., 2011).

## Effect of blue light exposure on retinal pigmented epithelium

The RPE forms a crucial interface between the neural retina and choroid, the vascular supply of the outer retina. The RPE controls the transportation of nutrients, ions, and water to photoreceptors, absorbs light and safeguards against photooxidation, converts all-trans-retinal to 11-cis-retinal to support the visual cycle, engulfs and discards photoreceptor membranes through phagocytosis, and secretes vital factors contributing to the structural integrity of the retina. The RPE is also important in combating lipid photooxidation and the generation of ROS, both of which are toxic to the retina, serving as the main defense system to counterbalance the high oxidative stress in the retina (Simó et al., 2010).

The outer blood-retinal barrier (oBRB), consisting of RPE monolayer and their tight junctions with Bruch’s membrane, controls nutrient and metabolite exchange vital for photoreceptor survival. Blue light exposure disrupts RPE tight junctions, resulting in loss of barrier function in rodents, rabbits and cultured human RPE cells. The decline in barrier function is associated with alterations in RPE tight junction proteins, specifically zonula occludens-1 (ZO-1) (Putting et al., 1992; Ozkaya et al., 2019; Xie et al., 2021). Blue light (400–520 nm) at 50 J/cm^2^energy (0.014 W/cm^2^ for 1 h) causes oBRB dysfunction in rabbits, 30 times more effectively than yellow light (510–740 nm) at the same energy (Putting et al., 1992, 1994). In human RPE cells, this effect is attributed to the dysfunction of Protein Kinase C-*ζ* (PKC-ζ), a component of a protein scaffold complex that regulates the polarity of RPE tight junctions (Ozkaya et al., 2019).

Morphological changes observed in cultured human RPE cells exposed to blue light include the presence of cracked nuclei, swollen mitochondria, disappearance of the inner limiting membrane of the mitochondria, vacuole formation, and dilation of the rough endoplasmic reticulum (Busch et al., 1999; Zhang, 2005; Roehlecke et al., 2009; Song et al., 2022; Françon et al., 2024). Blue light also impairs the secretion of retinal neurotrophic factors important for the maintenance of retinal cells such as hepatocyte growth factor and angiogenin by human RPE cells and vascular endothelial growth factor-A (VEGF-A) by porcine RPE cells (Chu et al., 2006; Vila et al., 2017; Marie et al., 2019). Transcriptome profiling by RNA-seq following blue light exposure of cultured human RPE cells demonstrated an upregulation of a broad range of genes involved not only in cell survival and cell cycle regulation, but also cell–cell interactions, cell morphology, inflammation and oxidative stress (Cheng et al., 2021).

Blue light can also cause a reversible inhibition of phagocytic activity and reduce receptor proteins, likely arising from oxidative modifications of phagocytic machinery in human RPE (Olchawa et al., 2022). The build-up of lipofuscin (a fluorophore that increases with age in RPE and results in increased oxidative stress) contributes to this reduced ability of cultured rabbit RPE cells to phagocytose the photoreceptor outer segments (Brunk et al., 1995; Sundelin et al., 1998). Loss of this critical function of RPE leads to photoreceptor dysfunction (Olchawa et al., 2017) (Figure 1). A more recent study in Japanese quail demonstrates that blue light exposure-induced photo-oxidative stress contributes to lipofuscin accumulation in RPE cells in the form of melanolipofuscin-like granules, which is suggested to impair RPE function (Serejnikova et al., 2024).

## Potential mechanisms of blue light-induced retinal damage

### Activation of inflammatory pathways

It is well established that blue light induces RPE to secrete cytokines and chemokines and growth factors. Specifically, blue light exposure results in an increased release of IL-6, IL-8, IL-17a and basic fibroblast growth factor (bFGF) in human RPE cells compared to other wavelengths (Sato et al., 2021). Additionally, chemokines involved in recruiting immune cells such as monocyte chemotactic protein-1(MCP-1) were also shown to be elevated in RPE-choroid complexes of mice exposed to blue light compared to other wavelengths (Narimatsu et al., 2015). This increase in cytokine secretion from blue light exposure has been shown to induce local proliferation and migration of activated immune cells such as microglia and macrophages in mice, which can contribute to further damage to the RPE (Figure 1) (Nakamura et al., 2018). Activation of these innate immune cells is not seen with white light (400–700 nm) exposure in mice, further highlighting the heightened effects of blue light on immune activation (Ebert et al., 2012).

### Oxidative stress

A key mechanism of blue light-induced RPE injury is the generation of ROS, resulting in oxidative damage and reduced viability through increased lipid peroxidation in cultured human and bovine RPE cells (Nakanishi-Ueda et al., 2013; Abdouh et al., 2022). The major source of ROS in human RPE is the mitochondria, particularly the endogenous fluorophores in the inner mitochondrial membrane (King et al., 2004). By making RPE more vulnerable to oxidative stress, blue light exposure can thus promote necroptosis, a form of programmed cell death that has features of both necrosis and apoptosis, and is independent of caspase activity in human RPE cells (Song et al., 2022). Antioxidant compounds such as lipoxins reduce RPE oxidative stress injury in cultured human RPE and in mice exposed to blue light (Xie et al., 2021).

RPE photoreactivity increases with age due to lipofuscin accumulation, which arises from incomplete degradation of photoreceptor outer segments, as observed in primary porcine, bovine and human RPE cells (Rozanowska et al., 1996; Marie et al., 2018). Lipofuscin toxicity is driven by bisretinoid fluorophores, which oxidize in response to oxygen and blue light, forming toxic aldehydes and ketones (Feldman et al., 2022). The main fluorophore, N-retinylidene-N-retinylethanolamine (A2E), accumulates with age in humans and acts as a photosensitizer, increasing ROS levels and RPE damage (Sparrow et al., 2000). A2E can also disrupt lysosomal membrane permeability, leading to mitochondrial damage, DNA damage, and apoptotic signaling (Xu et al., 2022) It triggers apoptosis by increasing calcium leakage from mitochondria and lysosomes into the cytosol (Brini et al., 2013), ultimately activating the mitochondrial apoptotic pathway.

### Mitochondrial dysfunction

Mitochondria play a crucial role in RPE damage from blue light exposure. At lower levels of blue light (1–3 mW/cm^2^), mitochondrial respiratory chain activity increases in RPE cells in Japanese quails (Serezhnikova et al., 2017). Human *in vitro* RPE models show a rise in mitochondria numbers and morphological changes, with larger, ring-shaped mitochondria forming, increasing membrane surface area. These adaptations likely enhance cellular resistance to ROS by boosting energy transfer efficiency and metabolic activity (Roehlecke et al., 2009).

At higher blue light intensities (>4 mW/cm^2^), the balance of mitochondrial fusion and fission is disrupted in mice and in cultured human RPE cells, leading to increased fragmentation (Anitua et al., 2023; Wang et al., 2023). This effect occurs in human RPE even without A2E loading (Alaimo et al., 2019). The fusion-fission balance is crucial for maintaining a healthy mitochondrial network, and its dysregulation marks an early step in apoptosis (McBride and Scorrano, 2013). Mitochondrial pathways, rather than purely ROS signaling, play a significant role in blue light-induced cell death in human RPE (Moon et al., 2017).

### Overactivation of autophagy

Autophagy is a protective mechanism in RPE that maintains cellular homeostasis by the degradation of harmful material from the ER, mitochondria or lysosomes (Intartaglia et al., 2022). However, autophagy competes with LC3-associated phagocytosis (LAP), a form of non-canonical autophagy that combines components of autophagy with phagocytosis to facilitate clearance of cellular debris. LAP is the primary process utilized by RPE to degrade the shed photoreceptor outer segments (POS), produce 11-cis retinal for the visual cycle and recycle essential components for photoreceptor disc renewal (Kim et al., 2013). Therefore, autophagy must be tightly regulated to ensure LAP occurs efficiently, prevent accelerated lipofuscin buildup, maintain POS clearance, and support the visual cycle (Kim et al., 2013; Ferguson and Green, 2014). Excessive autophagy activation has been observed in human and rodent RPE exposed to blue light, potentially due to the increased ROS and lysosomes and mitochondria permeability, disrupting the balance of autophagy and LAP. This imbalance reduces RPE viability and promotes lipofuscin buildup (Figure 1) (Fujita et al., 2007; Shen et al., 2013; Lee et al., 2015; Galluzzi et al., 2018).

## Experimental models of blue light stimulation

Experimental models are used to mimic environmental stressors associated with blue light stimulation through a variety of methods which are summarized below:

### *In vitro* models

The ARPE-19 cell line is the most commonly used cell model, along with primary RPE cell cultures. Study parameters such as exposure duration and irradiance intensity vary extensively in the literature (Table 1). *In vitro* experiments range from low intensity (0.04–3.7 mW/cm^2^) and long duration (3–48 h) (Roehlecke et al., 2009; Moon et al., 2017; Marie et al., 2018; Cheng et al., 2021; Anitua et al., 2023; Françon et al., 2024) to high intensity (4.4–100 mW/cm^2^) and short duration (1–90 min) (Pautler and BIOPHYSICS, 1990; Rozanowska et al., 1996; Sparrow and Cai, 2001; King et al., 2004; Alaimo et al., 2019; Abdouh et al., 2022; Olchawa et al., 2022). Despite these variations, similar effects on RPE have been observed. For example, Jeong *et al* exposed A2E-laden ARPE-19 to 6,000 lux blue light for 5 min/day for 120 days, while Burght *et al* used a single 15 h exposure of 1 mW/cm^2^ of blue light, yet both found similar changes in apoptosis and inflammation and complement-related gene expression (Van Der Burght et al., 2013; Jin and Jeong, 2022).

**Table 1:** Blue light wavelengths, irradiance and times of exposure from *in vitro* experimental studies reviewed here are compiled to compare and highlight the variability in the methodologies used.

Blue light wavelength (nm)	Irradiance (mW/cm^2^ or lux)	Time of exposure (hours or minutes)	Summary of methods and reference	Effects of blue light stimulation
455	0.185 J/cm^2^	0.305 h	Model used: Human induced pluripotent stem cells (hiPSC)-derived retinal pigment epithelial (iRPE) cells Light source: LED lamp blue (455 nm), white (3,300 K), or red (630 nm) light below phototoxicity thresholds (3.6 J/cm^2^ for white, 0.185 J/cm^2^ for blue, 0.276 J/cm^2^ for red). Reference: Françon et al. (2024)	Light below 22 J/cm^2^ induces structural changes, DNA damage, cellular stress, and alters autophagy in iRPE cells with blue light exposure. White light induces inflammation, while red light exhibits anti-inflammatory effects. The entire light spectrum significantly impacts RPE cell phototoxicity.
450	2.3 mW/cm^2^	6–24 h	Model used: ARPE-19 Light source: LED lamp Reference: Wang et al. (2023)	Prolonged blue light caused mitochondrial damage and dysfunction in RPE cells, disrupting mitochondrial dynamics with fusion-related blockage.
470	500 lux	24, 48 h	Model used: ARPE-19 cells Light source: LED lights Reference: Anitua et al. (2023)	Plasma rich in growth factors (PRGF) membrane with tailored optical properties provided protection against blue light-induced oxidative stress comparable to that of natural PRGF collected from healthy donors.
400–500	100 mW/cm^2^	0.5 h	Model used: Primary human RPE and ARPE-19 Light source: Solar simulator Reference: Abdouh et al. (2022)	Filtering blue light using yellow-tinted IOLs reduces oxidative stress and cell death caused by blue light exposure. Additionally, the antioxidant NAC protects RPE cells from blue light-induced ROS production, highlighting oxidative stress as a key factor in RPE damage.
390–510	18.1 mW/cm^2^	0.5, 1.0, 1.5 h	Model used: ARPE-19 cells lacking photoreactive pigments Light source: Solar simulator with band-pass (400–700 nm) and blue-light (410–500 nm) filters Reference: Olchawa et al. (2022)	Sublethal blue light hinders phagocytic activity in RPE cells. Blue light causes dose-dependent oxidation of cellular proteins and lipids, indicating vulnerability of RPE cells to phototoxic stress.
Not listed	2000 lux	6 h	Model used: Primary human RPE cells Light source: Not listed Reference: Luo et al. (2022)	Blue light elevates PKC activity, leading to RPE cell apoptosis through increased intracellular calcium. Chloroquine’s action on Bcl-2 proteins highlights their role in apoptosis inhibition in blue light-exposed RPE cells.
440	3.7 ± 0.75 mW/cm^2^, 0–639 J/cm^2^	0–48 h	Model used: ARPE-19 cells Light source: Blue-light-emitting diodes Reference: Cheng et al. (2021)	Blue light exposure causes damage to RPE via increase in apoptosis in a time-dependent manner. Oxidative stress at 2 h, DNA damage after 8 h and autophagy activation at 24-48 h of exposure. RNAseq data reveals that genes associated with tissue maturation, cell–cell intractions, movement, morphology and inflammation are altered.
450	2000, 1,000, 500, 250 lux	24 h	Model used: ARPE-19 cells Light source: 6500 K daylight-colored fluorescent lamp with blue filter Reference: Sato et al. (2021)	Continuous visible light exposure suppresses most cytokines but sustains VEGF-A levels and increases IL-17A and bFGF under blue light, correlating with light intensity. Anti-VEGF antibodies increase cytokine secretion of IL-6, IL-8, bFGF and MCP-1, potentially in response to VEGF suppression in irradiated RPE cells.
Not listed	2000 ± 500 lux	6 h	Model used: A2E-laden primary human RPE cells Light source: Not listed Reference: Luo et al. (2021)	Blue light exposure increased calcium levels in RPE cell cytoplasm, lysosomes, and mitochondria. A2E damaged lysosomal and mitochondrial membranes, releasing calcium into the cytoplasm. Both blue light and A2E reduced mitochondrial membrane potential, raising cytosolic calcium levels and promoting RPE cell death.
430	1,000 lux	15 h	Model used: A2E-laden ARPE-19 cells Light source: Not listed Reference: Xie et al. (2021)	Lipoxin A4, an endogenous lipid mediator mitigated oxidative stress and cell death in A2E-laden RPE cells exposed to blue light, and enhanced antioxidant enzyme expression (HO1, NQO1) via NRF2-Keap1 pathway modulation.
445 ± 18	4.43 mW/cm^2^	1–60 min	Model used: ARPE-19 cells and A2E-loaded ARPE-19 cells Light source: LED-based device Reference: Alaimo et al. (2019)	Blue light induced mitochondrial fragmentation by altering fusion/fission balance in both A2E-loaded and non-loaded cells. This imbalance correlated with changes in mitochondrial-shaping proteins (OPA1, DRP1, OMA1), indicating blue light exposure deregulates mitochondrial dynamics in RPE cells, contributing to cell death.
468	2.67 mW/cm^2^ 4.705 W/cm^2^ 7.465 W/cm^2^ 11.81 W/cm^2^	90 h	Model used: ARPE-19 and hTERT-RPE1 cell lines Light source: LED array circuit with 12 LEDs (Cree 5 mm Blue) Reference: Ozkaya et al. (2019)	Blue light reduces RPE barrier function and leads to cell death by over-activating PKC-*ζ* and causing oxidative stress. Inhibiting PKC-ζ may protect against blood-retinal barrier breakdown in AMD.
Not listed	Not listed	1 and 4 h	Model used: ARPE-19 and BEAS-2B cells Light source: Commercial light bulbs (incandescent, halogen, and LEDs of different color temperatures) Reference Gea et al. (2018)	Cold LED bulbs exhibited the most harmful effects, suggesting warmer LED options may be safer for retinal cells despite LED technology generally being safer than older lighting types.
390–520	1.5 mW/cm^2^	15 h	Model used: Porcine primary RPE cells cultured with A2E Light source: LED-based device with narrow light bands spanning 390 to 520 nm and a 630 nm band, mimicking solar spectrum conditions reaching the retina, focusing on harmful blue spectrum regions using precise 10 nm light bands. Reference: Marie et al. (2018)	415–455 nm blue–violet light is the solar spectrum wavelengths that triggers significant oxidative stress and mitochondrial dysfunction in A2E-exposed RPE cells.
449 458 470	0.04 W/(m^2^srnm)^−1^	24, 48 h	Model used: A2E-loaded ARPE-19 cells Light source: Display devices that emit blue light at specific wavelengths. Reference: Moon et al. (2017)	Even at the low intensity used in display devices, blue light can trigger ROS production and apoptosis in retinal cells.
460	80 lux	0–48 h	Model used: ARPE-19 and ATCC CRL-2302 RPE cells exposed to synthetic A2E Light source: LED plates Reference: Lin et al. (2017)	Low-luminance blue light, but not red light increases RPE apoptosis. Periodic blue light exposure induces Bax/Bcl-2, Fas/FasL pathways and caspase cascades in RPE cells.
470	1, 10, 50 J/cm^2^ (source is 4.8 mW/cm^2^)	3.5, 34.7, 173.6 min	Model used: Cultured bovine RPE cells Light source: custom LED system Reference: Nakanishi-Ueda et al. (2013)	Blue light exposure induces oxidative stress and cellular damage in RPE cells, even at relatively low doses (1–10 J/cm^2^), as evidenced by intracellular ROS generation, lipid peroxidation and loss of cell viability.
468	5 mW/cm^2^	3 cycles of 12 h	Model used: HRPEpiC human RPE cells Light source: LED-based system Reference: Eva Chamorro (2013)	Blue light filter reduced apoptosis by 56–89% and DNA damage by 57–81% in LED-exposed cells. It also lowered ROS production and increased cell viability in RPE cells exposed to LED light, indicating its photoprotective benefits against blue light-induced RPE damage.
430 ± 30	1 mW/cm^2^ 8 mW/cm^2^	7, 12, 20 min	Model used: ARPE-19 cells loaded with A2E Light source: 100 W mercury lamp Reference: Westlund et al. (2009)	c-Abl and p53 are essential for cell death in A2E-laden RPE cells under blue light exposure. The MAP kinase, JNK potentially acts protectively against apoptosis. Blocking c-Abl or p53 individually did not completely prevent cell death, suggesting multiple pathways are involved in phototoxicity.
405	0.3 mW/cm^2^ 1 mW/cm^2^	3, 24, 72 h	Model used: ARPE-19 cells Light source: LED-based system Reference: Roehlecke et al. (2009)	ARPE-19 cells activate stress response proteins (HO-1, Hsp-27, SOD-Mn etc.) and modify mitochondrial function to enhance resilience against low-dose non-lethal blue light irradiation.
488 514	500 mW/cm^2^	10 min	Model used: hTERT-RPE cells Light source: Argon-ion laser Reference: Glickman et al. (2005)	The study demonstrated that irradiation induces oxidative stress primarily due to melanin granules in RPE cells. It suggests that photooxidative stress in RPE cells leads to activation of NF-kB in RPE cells, which is alleviated by ascorbic acid treatment.
425 ± 20	1,000 mW/cm^2^	1 min	Model used: ARPE-19 cells Light source: 100 W mercury lamp Reference: King et al. (2004)	Mitochondria-derived ROS significantly contribute to RPE cell death from short-wavelength blue light. Targeting the mitochondrial electron transport chain or using mitochondria-specific antioxidants could potentially treat AMD by mitigating ROS and cell death.
430	19 mW/cm^2^	5–60 min	Model used: A2E-laden ARPE-19 cells Light source: Tungsten halogen source Reference Sparrow et al. (2003)	This study concludes that ROS generated from A2E interaction damages DNA, primarily through oxidative base modifications. Blue light exposure reduces DNA repair capacity proportional to exposure duration, impacting cellular repair processes.
480 ± 20 470 ± 20	3,500 mW/cm^2^ for 60 s 40 mW/cm^2^ for 20 min	1 min, 20 min	Model used: A2E-laden ARPE-19 cells Light source: 100 W mercury lamp or tungsten halogen source Reference: Sparrow and Cai (2001)	This study shows that blue light exposure to RPE cells containing intracellular A2E triggers a cell death pathway mediated by a proteolytic caspase cascade. Bcl-2 suppresses this pathway and thus suppresses apoptosis in RPE cells.
408–495	220 mW/cm^2^	Not listed	Model used: Primary human and bovine RPE cells Light source: High pressure xenon lamp Reference: Rozanowska et al. (1996)	Oxygen uptake in RPE cells varied by wavelength, peaking at 290 nm and decreasing significantly at 578 nm. Human RPE cells exhibited higher oxygen uptake than bovine cells, attributed to chromophore or melanin-related differences. Oxygen uptake increased with donor age in human RPE cells, while hydrogen peroxide formation showed modest changes under light exposure.
430	20 mW/cm^2^	1 h	Model used: Isolated bovine RPE Light source: Not listed Reference: Pautler and Colorado State Univ Fort Collins Department of Physiology and Biophysics (1990)	Blue light inhibits leucine, glutamate, and chloride flux from retina to choroid. Lower radiation levels show no effect. Neural retina-derived factors help maintain RPE. Blue light disrupts these transport systems, potentially contributing to AMD. Antioxidants (ascorbate, morin, melatonin, and vitamin E) do not alleviate blue light’s transport inhibition.

The wavelength of blue light also varies between studies. Blue light typically refers to wavelengths around 445 nm, the “Blue Light Hazard” (BLH) wavelength which causes photochemical damage (Van Norren and Gorgels, 2011). BLH refers to the damage caused by light with a polychromatic profile containing peaks at 445 nm, often leading to visible morphological damage (Françon et al., 2024). Wavelength variations affect results, and using LED light sources with known peaks at 445 nm is recommended to prevent inconsistencies in experimental studies. Some studies use white fluorescent lamps or solar simulators, but their peak wavelengths are often unknown, complicating accurate irradiance calculations. Although less common in *in vitro* models compared to *in vivo*, white fluorescent lamps with a blue-light filter or solar light simulators may be used (Gea et al., 2018; Sato et al., 2021; Olchawa et al., 2022). Narrow-band interference filters or blue film filters allow for light exposure in the range of 400-490 nm, broad-band pass filters provide a range of 400-520 nm or 400-700 nm, and UV and infrared (IR) blocking filters remove wavelengths below 400 nm and above 740 nm, respectively (Putting et al., 1994; Olchawa et al., 2022). Neutral-density filters are used to standardize the intensity of light without altering its spectral composition (Ozkaya et al., 2019; Sato et al., 2021). However, the peak wavelength of these light sources is often unknown, leading to difficulties in accurate calculation of their irradiance intensities. Therefore, light sources with more uniform peak wavelengths such as LED devices should be utilized to prevent variance of wavelengths in blue light studies.

A common baseline for irradiance intensity is the phototoxic threshold, which is the threshold of irradiance intensity at which microscopic phototoxic damage occurs. The phototoxic threshold for blue light at 445 nm in humans was initially determined to be 22 J/cm^2^ (Van Norren and Gorgels, 2011). However, this threshold of the BLH may have been overestimated, as phototoxic damage has been observed in animal models at intensities lower than the estimated threshold by a factor of 20 (Hunter et al., 2012; Jaadane et al., 2020). Sub-threshold exposure in human *in vitro* RPE models also shows phototoxic changes in RPE morphology and immunological responses, suggesting that lower irradiance levels should be further explored (Françon et al., 2024).

A majority of studies measure blue light intensity in mW/cm^2^, which measures the intensity per unit area, but not the total amount of light energy received by the cells. This is problematic since exposure duration varies between studies, making it challenging to compare results. Measuring the total energy (J/cm^2^) would better reflect the cumulative light exposure, enabling easier comparisons across studies (Van Norren and Gorgels, 2011). Furthermore, the use of lux, a measure of illuminance, instead of mW/cm^2^, adds another layer of complexity, as lux measures light emitted rather than received. Therefore, providing mW/cm^2^, duration and total energy (J/cm^2^) in studies will allow for more consistent comparisons across studies.

### *In vivo* and *ex vivo* models

While cell-based blue light experiments are easier to design and provide more reliable, reproducible results, *in vivo* models offer significant advantages over cultured RPE cells: (1) Animal studies offer more physiologically relevant insights into the complex structural and functional interactions between different cell types in retinal tissue which are often lacking in cultured systems, since these model systems preserve systemic responses such as neuronal activity, blood circulation and immune activation. (2) Cells in the neural retina such as photoreceptors lose their morphological integrity *in vitro*. This issue can be addressed by using *in vivo* or *ex vivo* models to study blue light-mediated effects on the retina and its interactions with the RPE/choroid in a more complex physiologic environment. (3) *In vivo* models also allow longitudinal studies for studying the effects of low intensity, long-term blue light exposure and tracking changes over time in the same animal.

*Ex vivo* models containing neural retina, RPE-Bruch’s membrane-choroid complexes or whole eyes can be used to assess chronic or intermittent blue light effects on ocular tissue under controlled conditions. One study utilized whole eyeball cultures to examine the impact of blue light exposure on mouse photoreceptors (Roehlecke et al., 2011). Eyeballs were punctured with a needle to enable fluid exchange and maintained in serum-containing medium at 37°C and 5%CO_2_. The eyes were positioned with corneas facing the blue light diodes and were irradiated from 0.5–24 h. In another study, whole porcine eyes were irradiated with blue light for 3 h and incubated for further 6 h in PBS. Isolated neural retina was also exposed to blue light for 1–2 h and maintained on cell culture inserts for 24–48 h (Fietz et al., 2023). Standardization of *ex vivo* models would greatly benefit the field of blue light study. Additionally, *ex vivo* models used for studying choroidal microvascular angiogenesis could be adapted for blue light research. Rodent or human RPE/choroid/scleral tissue can be readily isolated and cultured for up to 6 days, allowing for reproducible evaluation of specific pathways involved in blue light-induced responses (Shao et al., 2013; Tomita et al., 2020). Such explants can also be used to compare differences between genetically modified mouse tissue and wild type following blue light stimulation.

One limitation of rodent models for AMD and blue light research is the absence of a macula, an area critical to high-resolution vision in humans. Another challenge with using animal models is the inherent variability in irradiance intensities on the ocular tissue (Table 2) (Narimatsu et al., 2015; Lin et al., 2017; Xie et al., 2021; Wang et al., 2023). *Ex vivo* models allow a more precise manipulation of illumination conditions, compared to animal models, thereby improving reproducibility and reducing complexity due to systemic influences. Despite some limitations, both *in vivo* and *ex vivo* model systems provide significant advantages.

**Table 2:** Blue light wavelengths, irradiance and times of exposure from *in vivo* experimental studies reviewed here are compiled to compare and highlight the variability in the methodologies used.

Blue light wavelength (nm)	Irradiance (mW/cm^2^ or lux)	Time of exposure (hours or minutes)	Summary of methods and reference	Effects of blue light stimulation
450	4 J/ cm^2^	40 min	Model used: Japanese quail Light source: Blue LED Reference: Serejnikova et al. (2024)	Photo-oxidative stress due to blue light leads to active fusion of melanosomes and lipofuscin granules, forming melanolipofuscin-like granules in RPE cells.
450	800 lux	336 h	Model used: 6-month-old C57BL/6 mice Light source: LED lamp Reference: Wang et al. (2023)	Prolonged blue light damaged the outer nuclear layer and RPE cells in mice. It also caused mitochondrial damage and dysfunction in RPE cells, disrupting mitochondrial dynamics with fusion-related blockage.
430	10,000 lux	1 h each day for 14 days	Model used: Male Balb-c mice Light source: Not listed Reference: Xie et al. (2021)	Lipoxin A4, an endogenous lipid mediator preserved retinal health by shielding RPE cells from structural and functional damage in a mouse model of blue light-induced retinal degeneration.
440	3.7 ± 0.75 mW/cm^2^, 0–639 J/cm^2^	24, 30 h	Model used: zebrafish larvae Light source: Blue-light-emitting diodes Reference: Cheng et al. (2021)	Blue light exposure appears to have an unfavorable effect on retinal tissue development. Blue light reduced thickness of all retinal layers, induced cytotoxicity (increased TUNEL and caspase-3 staining) in retinal cells including RPE cells.
455–470	5.03 lux; 0.0123 mW/cm^2^	3, 6, 12 h	Model used: Sprague–Dawley rats Light source: custom-built blue light illuminator from analog cell phone array Reference: Li et al. (2021)	Long term exposure to low-illuminance blue light causes retinal tissue structure and functional damage. Photoreceptor amplitude decreased, peak times delayed, RPE layer thinned, photoreceptor membrane discs damaged.
456	1,100 lux	3, 9 h	Model used: Male C57BL/6 J mice Light source: LED lamp Reference: Nakamura et al. (2018)	Three days of blue LED exposure caused macrophage buildup, drusen-like material at the photoreceptor junction, initial RPE cell enlargement, and subsequent photoreceptor degeneration. This damage differed from effects seen with white light.
460	150 lux	0, 0.5, 1, 3 h	Model used: Brown Norway (BN) rats with intravitreal A2E injections Light source: LED plates Reference: Lin et al. (2017)	Low-luminance blue light worsens A2E-induced phototoxicity, damaging the retina. Blue light exposure reduces fundus integrity, retinal thickness, and disrupts retinal neuron function in rats. Combined A2E and periodic blue light exposure markedly decrease retinal thickness and photoreceptor layers, exacerbating toxicity to RPE.
440–460	2 mW/cm^2^	15 h	Model used: Japanese quails Light source: LED lights Reference: Serezhnikova et al. (2017)	Young birds exposed to daily blue light exhibited increased total and altered mitochondria in RPE cells. Adult birds showed enhanced metabolic activity in RPE cells after blue light exposure, indicating a mitochondria-driven response to mitigate blue light-induced damage and lipofuscin accumulation in the RPE.
420, 446	3,000 lux	20 min	Model used: 7–8 weeks old male BALB/c mice light-adapted with 12 h of darkness Light source: White fluorescent lamp within a mirrored light-exposure chamber Reference: Narimatsu et al. (2015)	Yellow intraocular lens effectively suppresses light-induced ROS levels, inflammatory cytokine expression, and macrophage recruitment in RPE-choroid complexes of mice. Blocking blue light can mitigate ROS accumulation and potentially lower the risk of choroidal neovascularization (CNV) *in vivo.*
410	8.7 mW/cm^2^	2 min	Model used: MacGreen mice, expressing eGFP under the Csf1r promoter Light source: Xenon arc reflector lamp Reference: Ebert et al. (2012)	Blue light exposure led to microglial proliferation and migration towards retinal lesions, adopting activated amoeboid morphology. Transcriptomic changes seen in microglial activation, apoptosis and cell survival genes.
400–520	62 to 832 J/cm^2^	12 h	Model used: New Zealand albino rabbits and pigmented chinchilla rabbits Light source: 1000 W xenon arc lamp Reference Putting et al. (1994)	Blue light at 439 *±* 6 nm was more effective than other wavelengths in inducing blood-retinal barrier dysfunction in albino rabbits. Melanin in RPE cells does not have an effect on blue light-induced phototoxicity.
400–520	14 mW/cm^2^	1 h	Model used: Rabbit retinas Light source: 1000 W xenon arc lamp Reference: Putting et al. (1992)	The results demonstrate that the blue component of white light causes dysfunction of the blood-retinal barrier at the RPE 30 times more effectively than the longer wavelength fraction of white light.

## Conclusion and future perspectives

In conclusion, while some evidence suggests a potential association between prolonged blue light exposure and increased AMD risk, more epidemiological studies are needed to establish a definitive link. Blue light induces oxidative stress, disrupts cell structures, and impairs essential RPE functions, leading to apoptosis and the AMD progression. Further research could also focus on protective strategies, including therapeutic interventions targeting pathways activated by blue light and improving blue-filtering technology to prevent retinal damage. Moreover, refining blue light exposure experiments—through standardized protocols, precise light irradiance measurements, and the development of novel *in vivo* and *ex vivo* models—will improve the consistency and relevance of findings across studies and enhance our understanding of blue light’s effects on the retina and ocular tissues.

## References

[ref1] Abdouh M. Lu M. Chen Y. Goyeneche A. Burnier J. V. Burnier M. N. (2022). Filtering blue light mitigates the deleterious effects induced by the oxidative stress in human retinal pigment epithelial cells. Exp. Eye Res. 217:108978. doi: 10.1016/j.exer.2022.108978, PMID: 35134392

[ref2] Achiron A. Elbaz U. Hecht I. Spierer O. Einan-Lifshitz A. Karesvuo P.. (2021). The effect of blue-light filtering intraocular lenses on the development and progression of Neovascular age-related macular degeneration. Ophthalmology 128, 410–416. doi: 10.1016/j.ophtha.2020.07.039, PMID: 32717342

[ref3] Alaimo A. Liñares G. G. Bujjamer J. M. Gorojod R. M. Alcon S. P. Martínez J. H.. (2019). Toxicity of blue led light and A2E is associated to mitochondrial dynamics impairment in ARPE-19 cells: implications for age-related macular degeneration. Arch. Toxicol. 93, 1401–1415. doi: 10.1007/s00204-019-02409-6, PMID: 30778631

[ref4] Anitua E. Muruzabal F. De La Fuente M. Del Olmo-Aguado S. Alkhraisat M. H. Merayo-Lloves J. (2023). PRGF membrane with tailored optical properties preserves the Cytoprotective effect of plasma rich in growth factors: in vitro model of retinal pigment epithelial cells. Int. J. Mol. Sci. 24:11195. doi: 10.3390/ijms241311195, PMID: 37446374 PMC10342881

[ref5] Baker J. Putnam N. Kozlowski R. E. Anderson M. Bird Z. Chmielewski J.. (2022). Effects of chronic, daily exposures to low intensity blue light on human retinal pigment epithelial cells: implications for the use of personal electronic devices. J. Photochem. Photobiol. 10:100118. doi: 10.1016/j.jpap.2022.100118

[ref6] Behar-Cohen F. Martinsons C. Viénot F. Zissis G. Barlier-Salsi A. Cesarini J. P.. (2011). Light-emitting diodes (LED) for domestic lighting: any risks for the eye? Prog. Retin. Eye Res. 30, 239–257. doi: 10.1016/j.preteyeres.2011.04.002, PMID: 21600300

[ref7] Brini M. Calì T. Ottolini D. Carafoli E. (2013). The plasma membrane calcium pump in health and disease. FEBS J. 280, 5385–5397. doi: 10.1111/febs.12193, PMID: 23413890

[ref8] Brunk U. T. Wihlmark U. Wrigstad A. Roberg K. Nilsson S.-E. (1995). Accumulation of lipofuscin within retinal pigment epithelial cells results in enhanced sensitivity to photo-oxidation. Gerontology 41, 201–212. doi: 10.1159/000213743, PMID: 8821332

[ref9] Busch E. M. Gorgels T. G. M. F. Van Norren D. (1999). Temporal sequence of changes in rat retina after UV-A and blue light exposure. Vis. Res. 39, 1233–1247. doi: 10.1016/S0042-6989(98)00233-8, PMID: 10343838

[ref10] Cheng K.-C. Hsu Y.-T. Liu W. Huang H.-L. Chen L.-Y. He C.-X.. (2021). The role of oxidative stress and autophagy in blue-light-induced damage to the retinal pigment epithelium in zebrafish in vitro and in vivo. Int. J. Mol. Sci. 22:1338. doi: 10.3390/ijms22031338, PMID: 33572787 PMC7866289

[ref11] Chu R. Zheng X. Chen D. Hu D. (2006). Blue light irradiation inhibits the production of HGF by human retinal pigment epithelium cells in vitro. Photochem. Photobiol. 82, 1247–1250. doi: 10.1562/2006-04-19-RA-880, PMID: 16740060

[ref12] Contín M. A. Benedetto M. M. Quinteros-Quintana M. L. Guido M. E. (2016). Light pollution: the possible consequences of excessive illumination on retina. Eye 30, 255–263. doi: 10.1038/eye.2015.221, PMID: 26541085 PMC4763120

[ref13] Cougnard-Gregoire A. Merle B. M. J. Aslam T. Seddon J. M. Aknin I. Klaver C. C. W.. (2023). Blue light exposure: ocular hazards and prevention—a narrative review. Ophthalmol. Ther. 12, 755–788. doi: 10.1007/s40123-023-00675-3, PMID: 36808601 PMC9938358

[ref14] Cruickshanks K. J. Klein R. Klein B. E. Nondahl D. M. (2001). Sunlight and the 5-year incidence of early age-related maculopathy: the beaver dam eye study. Arch. Ophthalmol. Chic. Ill 1960, 246–250. 11176987

[ref15] Ebert S. Walczak Y. Remé C. Langmann T. (2012). Microglial activation and transcriptomic changes in the blue light-exposed mouse retina, 619–632. doi: 10.1007/978-1-4614-0631-0_79 22183386

[ref16] Eva Chamorro S. F. C. (2013). Photoprotective effects of blue light absorbing filter against LED light exposure on human retinal pigment epithelial cells in vitro. J. Carcinog. Mutagen. 1–7. doi: 10.4172/2157-2518.S6-00825177526

[ref17] Evans J. R. (2005). 28 000 cases of age related macular degeneration causing visual loss in people aged 75 years and above in the United Kingdom may be attributable to smoking. Br. J. Ophthalmol. 89, 550–553. doi: 10.1136/bjo.2004.049726, PMID: 15834082 PMC1772624

[ref18] Feldman T. Ostrovskiy D. Yakovleva M. Dontsov A. Borzenok S. Ostrovsky M. (2022). Lipofuscin-mediated photic stress induces a dark toxic effect on ARPE-19 cells. Int. J. Mol. Sci. 23:12234. doi: 10.3390/ijms232012234, PMID: 36293088 PMC9602730

[ref19] Ferguson T. A. Green D. R. (2014). Autophagy and phagocytosis converge for better vision. Autophagy 10, 165–167. doi: 10.4161/auto.26735, PMID: 24220227 PMC4028322

[ref20] Fietz A. Corsi F. Hurst J. Schnichels S. (2023). Blue light damage and p53: unravelling the role of p53 in oxidative-stress-induced retinal apoptosis. Antioxidants 12:2072. doi: 10.3390/antiox12122072, PMID: 38136192 PMC10740515

[ref21] Fletcher A. E. (2008). Sunlight exposure, antioxidants, and age-related macular degeneration. Arch. Ophthalmol. 126:1396. doi: 10.1001/archopht.126.10.1396 18852418

[ref22] Françon A. Delaunay K. Jaworski T. Lebon C. Picard E. Youale J.. (2024). Phototoxicity of low doses of light and influence of the spectral composition on human RPE cells. Sci. Rep. 14:6839. doi: 10.1038/s41598-024-56980-9, PMID: 38514646 PMC10957882

[ref23] Fujita E. Kouroku Y. Isoai A. Kumagai H. Misutani A. Matsuda C.. (2007). Two endoplasmic reticulum-associated degradation (ERAD) systems for the novel variant of the mutant dysferlin: ubiquitin/proteasome ERAD(I) and autophagy/lysosome ERAD(II). Hum. Mol. Genet. 16, 618–629. doi: 10.1093/hmg/ddm002, PMID: 17331981

[ref24] Galluzzi L. Vitale I. Aaronson S. A. Abrams J. M. Adam D. Agostinis P.. (2018). Molecular mechanisms of cell death: recommendations of the nomenclature committee on cell death 2018. Cell Death Differ. 25, 486–541. doi: 10.1038/s41418-017-0012-4, PMID: 29362479 PMC5864239

[ref25] Gea M. Schilirò T. Iacomussi P. Degan R. Bonetta S. Gilli G. (2018). Cytotoxicity and genotoxicity of light emitted by incandescent, halogen, and LED bulbs on ARPE-19 and BEAS-2B cell lines. J. Toxicol. Environ. Health A 81, 998–1014. doi: 10.1080/15287394.2018.1510350, PMID: 30325709

[ref26] Glickman R. D. Natarajan M. Rockwell B. A. Denton M. Maswadi S. Kumar N.. (2005) in Intracellular signaling mechanisms responsive to laser-induced photochemical and thermal stress. eds. Jacques S. L. Roach W. P..

[ref27] Hall H. Ma J. Shekhar S. Leon-Salas W. D. Weake V. M. (2018). Blue light induces a neuroprotective gene expression program in Drosophila photoreceptors. BMC Neurosci. 19:43. doi: 10.1186/s12868-018-0443-y, PMID: 30029619 PMC6053765

[ref28] Ham W. T. (1983). Ocular hazards of light sources: review of current knowledge. J. Occup. Med. 25, 101–103, PMID: 6834158

[ref29] Ham W. T. Mueller H. A. Ruffolo J. J. Clarke A. M. (1979). Sensitivity of the retina to radiation damage as a function of wavelength. Photochem. Photobiol. 29, 735–743. doi: 10.1111/j.1751-1097.1979.tb07759.x 109869

[ref30] Hunter J. J. Morgan J. I. W. Merigan W. H. Sliney D. H. Sparrow J. R. Williams D. R. (2012). The susceptibility of the retina to photochemical damage from visible light. Prog. Retin. Eye Res. 31, 28–42. doi: 10.1016/j.preteyeres.2011.11.001, PMID: 22085795 PMC3242847

[ref31] Intartaglia D. Giamundo G. Conte I. (2022). Autophagy in the retinal pigment epithelium: a new vision and future challenges. FEBS J. 289, 7199–7212. doi: 10.1111/febs.16018 33993621 PMC9786786

[ref32] Jaadane I. Villalpando Rodriguez G. Boulenguez P. Carré S. Dassieni I. Lebon C.. (2020). Retinal phototoxicity and the evaluation of the blue light hazard of a new solid-state lighting technology. Sci. Rep. 10:6733. doi: 10.1038/s41598-020-63442-5, PMID: 32317708 PMC7174369

[ref33] Jin H. L. Jeong K. W. (2022). Transcriptome analysis of long-term exposure to blue light in retinal pigment epithelial cells. Biomol. Ther. 30, 291–297. doi: 10.4062/biomolther.2021.155, PMID: 35074938 PMC9047491

[ref34] Jonasson F. Fisher D. E. Eiriksdottir G. Sigurdsson S. Klein R. Launer L. J.. (2014). Five-year incidence, progression, and risk factors for age-related macular degeneration. Ophthalmology 121, 1766–1772. doi: 10.1016/j.ophtha.2014.03.013, PMID: 24768241 PMC4145014

[ref35] Kim S. H. Kim Y. K. Shin Y. I. Kang G. Kim S. P. Lee H.. (2024). Nighttime outdoor artificial light and risk of age-related macular degeneration. JAMA Netw. Open 7:e2351650. doi: 10.1001/jamanetworkopen.2023.51650, PMID: 38227312 PMC10792474

[ref36] Kim J.-Y. Zhao H. Martinez J. Doggett T. A. Kolesnikov A. V. Tang P. H.. (2013). Noncanonical autophagy promotes the visual cycle. Cell 154, 365–376. doi: 10.1016/j.cell.2013.06.012, PMID: 23870125 PMC3744125

[ref37] King A. Gottlieb E. Brooks D. G. Murphy M. P. Dunaief J. L. (2004). Mitochondria-derived reactive oxygen species mediate blue light-induced death of retinal pigment epithelial cells ^¶^. Photochem. Photobiol. 79, 470–475. doi: 10.1562/le-03-17.1, PMID: 15191057

[ref38] Kuse Y. Ogawa K. Tsuruma K. Shimazawa M. Hara H. (2014). Damage of photoreceptor-derived cells in culture induced by light emitting diode-derived blue light. Sci. Rep. 4:5223. doi: 10.1038/srep05223, PMID: 24909301 PMC4048889

[ref39] Lambert N. G. ElShelmani H. Singh M. K. Mansergh F. C. Wride M. A. Padilla M.. (2016). Risk factors and biomarkers of age-related macular degeneration. Prog. Retin. Eye Res. 54, 64–102. doi: 10.1016/j.preteyeres.2016.04.003, PMID: 27156982 PMC4992630

[ref40] Lee W.-S. Yoo W.-H. Chae H.-J. (2015). ER stress and autophagy. Curr. Mol. Med. 15, 735–745. doi: 10.2174/1566524015666150921105453 26391548

[ref41] Li H. Zhang M. Wang D. Dong G. Chen Z. Li S.. (2021). Blue light from cell phones can cause chronic retinal light injury: the evidence from a clinical observational study and a SD rat model. Biomed. Res. Int. 2021, 1–13. doi: 10.1155/2021/3236892, PMID: 34055970 PMC8147535

[ref42] Lin C.-H. Wu M.-R. Li C.-H. Cheng H.-W. Huang S.-H. Tsai C.-H.. (2017). Editor’s highlight: periodic exposure to smartphone-mimic low-luminance blue light induces retina damage through Bcl-2/BAX-dependent apoptosis. Toxicol. Sci. 157, 196–210. doi: 10.1093/toxsci/kfx030, PMID: 28184904

[ref43] Luo M.-M. Chen L. Wang S. Zeng C. Li D.-Z. Bi Y.. (2021). The effect of A2E on the uptake and release of calcium in the lysosomes and mitochondria of human RPE cells exposed to blue light. J. Ophthalmol. 2021, 1–10. doi: 10.1155/2021/5586659, PMID: 34603771 PMC8486552

[ref44] Luo M. Wang S. Tang Y. Zeng C. Cai S. (2022). The effect of A2E on the Ca2+-PKC signaling pathway in human RPE cells exposed to blue light. J. Ophthalmol. 2022, 1–7. doi: 10.1155/2022/2233223, PMID: 36304713 PMC9596233

[ref45] Mallet J. D. Rochette P. J. (2011). Ultraviolet light-induced Cyclobutane pyrimidine dimers in rabbit eyes. Photochem. Photobiol. 87, 1363–1368. doi: 10.1111/j.1751-1097.2011.00977.x 21770949

[ref46] Mallet J. D. Rochette P. J. (2013). Wavelength-dependent ultraviolet induction of cyclobutane pyrimidine dimers in the human cornea. Photochem. Photobiol. Sci. 12, 1310–1318. doi: 10.1039/c3pp25408a, PMID: 23364620

[ref47] Marie M. Bigot K. Angebault C. Barrau C. Gondouin P. Pagan D.. (2018). Light action spectrum on oxidative stress and mitochondrial damage in A2E-loaded retinal pigment epithelium cells. Cell Death Dis. 9:287. doi: 10.1038/s41419-018-0331-5, PMID: 29459695 PMC5833722

[ref48] Marie M. Gondouin P. Pagan D. Barrau C. Villette T. Sahel J.. (2019). Blue-violet light decreases VEGFa production in an in vitro model of AMD. PLoS One 14:e0223839. doi: 10.1371/journal.pone.0223839, PMID: 31644596 PMC6808507

[ref49] McBride H. Scorrano L. (2013). Mitochondrial dynamics and physiology. Biochim. Biophys. Acta BBA Mol. Cell Res. 1833, 148–149. doi: 10.1016/j.bbamcr.2012.11.001 23142418

[ref50] Molday R. S. Moritz O. L. (2015). Photoreceptors at a glance. J. Cell Sci. 128, 4039–4045. doi: 10.1242/jcs.175687, PMID: 26574505 PMC4712787

[ref51] Moon J. Yun J. Yoon Y. D. Park S.-I. Seo Y.-J. Park W.-S.. (2017). Blue light effect on retinal pigment epithelial cells by display devices. Integr. Biol. 9, 436–443. doi: 10.1039/c7ib00032d, PMID: 28386617

[ref52] Nakamura M. Yako T. Kuse Y. Inoue Y. Nishinaka A. Nakamura S.. (2018). Exposure to excessive blue LED light damages retinal pigment epithelium and photoreceptors of pigmented mice. Exp. Eye Res. 177, 1–11. doi: 10.1016/j.exer.2018.07.022, PMID: 30040948

[ref53] Nakanishi-Ueda T. Majima H. J. Watanabe K. Ueda T. Indo H. P. Suenaga S.. (2013). Blue LED light exposure develops intracellular reactive oxygen species, lipid peroxidation, and subsequent cellular injuries in cultured bovine retinal pigment epithelial cells. Free Radic. Res. 47, 774–780. doi: 10.3109/10715762.2013.829570, PMID: 23898883

[ref54] Narimatsu T. Negishi K. Miyake S. Hirasawa M. Osada H. Kurihara T.. (2015). Blue light-induced inflammatory marker expression in the retinal pigment epithelium-choroid of mice and the protective effect of a yellow intraocular lens material in vivo. Exp. Eye Res. 132, 48–51. doi: 10.1016/j.exer.2015.01.003, PMID: 25576667

[ref55] Noell W. K. (1980). Possible mechanisms of photoreceptor damage by light in mammalian eyes. Vis. Res. 20, 1163–1171. doi: 10.1016/0042-6989(80)90055-3 7269272

[ref56] Noell W. K. Walker V. S. Kang B. S. Berman S. (1966). Retinal damage by light in rats. Invest. Ophthalmol. Vis. Sci. 5, 450–473. 5929286

[ref57] Olchawa M. M. Furso J. A. Szewczyk G. M. Sarna T. J. (2017). Lipofuscin-mediated photic stress inhibits phagocytic activity of ARPE-19 cells; effect of donors’ age and antioxidants. Free Radic. Res. 51, 799–811. doi: 10.1080/10715762.2017.1380307, PMID: 28969450

[ref58] Olchawa M. M. Herrnreiter A. M. Skumatz C. M. B. Krzysztynska-Kuleta O. I. Mokrzynski K. T. Burke J. M.. (2022). The inhibitory effect of blue light on phagocytic activity by ARPE-19 cells. Photochem. Photobiol. 98, 1110–1121. doi: 10.1111/php.13596, PMID: 35067943

[ref59] Ozkaya E. K. Anderson G. Dhillon B. Bagnaninchi P.-O. (2019). Blue-light induced breakdown of barrier function on human retinal epithelial cells is mediated by PKC-ζ over-activation and oxidative stress. Exp. Eye Res. 189:107817. doi: 10.1016/j.exer.2019.107817, PMID: 31563609

[ref60] Parkinson L. G. Toro A. Zhao H. Brown K. Tebbutt S. J. Granville D. J. (2015). Granzyme B mediates both direct and indirect cleavage of extracellular matrix in skin after chronic low-dose ultraviolet light irradiation. Aging Cell 14, 67–77. doi: 10.1111/acel.12298, PMID: 25495009 PMC4326907

[ref61] Pautler E. L. Colorado State Univ Fort Collins Department of Physiology and Biophysics. (1990). The Phototoxicity of’Blue Light’on the functional properties of the retinal pigment epithelium.

[ref62] Putting B. J. Van Best J. A. Vrensen G. F. J. M. Oosterhuis J. A. (1994). Blue-light-induced dysfunction of the blood-retinal barrier at the pigment epithelium in albino versus pigmented rabbits. Exp. Eye Res. 58, 31–40. doi: 10.1006/exer.1994.1192, PMID: 8157099

[ref63] Putting B. Zweypfenning R. Vrensen G. F. J. M. Oosterhuis J. A. Best J. A. V. (1992). Blood-retinal barrier dysfunction at the pigment epithelium induced by blue light. Invest. Ophthalmol. Vis. Sci. Available at: https://www.semanticscholar.org/paper/Blood-retinal-barrier-dysfunction-at-the-pigment-by-Putting-Zweypfenning/0c16c6f69b1f85e7a4ae0d49a7939ce403de8fd7 33, 3385–3393, PMID: 1428711

[ref64] Roehlecke C. Schaller A. Knels L. Funk R. H. W. (2009). The influence of sublethal blue light exposure on human RPE cells. Mol. Vis. 15, 1929–1938, PMID: 19784391 PMC2751800

[ref65] Roehlecke C. Schumann U. Ader M. Knels L. Funk R. H. W. (2011). Influence of blue light on photoreceptors in a live retinal explant system. Mol. Vis. 17, 876–884, PMID: 21527999 PMC3081800

[ref66] Rozanowska M. Jarvis-Evans J. Korytowski W. Boulton M. E. Burke J. M. Sarna T. (1996). Blue light-induced reactivity of retinal age pigment. Retina 16:354. doi: 10.1097/00006982-199616040-00023 7642534

[ref67] Sato T. Takeuchi M. Karasawa Y. Ito M. (2021). Profiles of cytokines secreted by ARPE-19 cells exposed to light and incubated with anti-VEGF antibody. Biomedicines 9:1333. doi: 10.3390/biomedicines9101333, PMID: 34680450 PMC8533158

[ref68] Schick T. Ersoy L. Lechanteur Y. T. E. Saksens N. T. M. Hoyng C. B. Den Hollander A. I.. (2016). History of sunlight exposure is a risk factor for age-related macular degeneration. Retina 36, 787–790. doi: 10.1097/IAE.0000000000000756, PMID: 26441265

[ref69] Serejnikova N. B. Trofimova N. N. Yakovleva M. A. Dontsov A. E. Zak P. P. Ostrovsky M. A. (2024). Blue light-induced accelerated formation of Melanolipofuscin-like organelles in Japanese quail RPE cells: an Electron microscopic study. Invest. Ophthalmol. Vis. Sci. 65:31. doi: 10.1167/iovs.65.11.31, PMID: 39297806 PMC11421679

[ref70] Serezhnikova N. B. Pogodina L. S. Lipina T. V. Trofimova N. N. Gurieva T. S. Zak P. P. (2017). Age-related adaptive responses of mitochondria of the retinal pigment epithelium to the everyday blue LED lighting. Dokl. Biol. Sci. 475, 141–143. doi: 10.1134/S0012496617040044, PMID: 28861875

[ref71] Shao Z. Friedlander M. Hurst C. G. Cui Z. Pei D. T. Evans L. P.. (2013). Choroid sprouting assay: an ex vivo model of microvascular angiogenesis. PLoS One 8:e69552. doi: 10.1371/journal.pone.0069552, PMID: 23922736 PMC3724908

[ref72] Shen K. Sun L. Zhang H. Xu Y. Qian X. Lu Y.. (2013). A ROS-mediated lysosomal-mitochondrial pathway is induced by a novel Amonafide analogue, 7c, in human Hela cervix carcinoma cells. Cancer Lett. 333, 229–238. doi: 10.1016/j.canlet.2013.01.038 23376642

[ref73] Simó R. Villarroel M. Corraliza L. Hernández C. Garcia-Ramírez M. (2010). The retinal pigment epithelium: something more than a constituent of the blood-retinal barrier—implications for the pathogenesis of diabetic retinopathy. J. Biomed. Biotechnol. 2010, 1–15. doi: 10.1155/2010/190724, PMID: 20182540 PMC2825554

[ref74] Song W. Zhu R. Gao W. Xing C. Yang L. (2022). Blue light induces RPE cell necroptosis, which can be inhibited by minocycline. Front. Med. 9, 1–14. doi: 10.3389/fmed.2022.831463, PMID: 35559340 PMC9086715

[ref75] Sparrow J. R. Cai B. (2001). Blue light-induced apoptosis of A2E-containing RPE: involvement of caspase-3 and protection by Bcl-2. Invest. Ophthalmol. Vis. Sci. 42, 1356–1362, PMID: 11328751

[ref76] Sparrow J. R. Nakanishi K. Parish C. A. (2000). The lipofuscin fluorophore A2E mediates blue light-induced damage to retinal pigmented epithelial cells. Invest. Ophthalmol. Vis. Sci. 41, 1981–1989, PMID: 10845625

[ref77] Sparrow J. R. Zhou J. Cai B. (2003). DNA is a target of the photodynamic effects elicited in A2E-laden RPE by blue-light illumination. Investig. Opthalmology Vis. Sci. 44, 2245–2251. doi: 10.1167/iovs.02-0746, PMID: 12714667

[ref78] Steinmetz J. D. Bourne R. R. A. Briant P. S. Flaxman S. R. Taylor H. R. B. Jonas J. B.. (2021). Causes of blindness and vision impairment in 2020 and trends over 30 years, and prevalence of avoidable blindness in relation to VISION 2020: the right to sight: an analysis for the global burden of disease study. Lancet Glob. Health 9, e144–e160. doi: 10.1016/S2214-109X(20)30489-7, PMID: 33275949 PMC7820391

[ref79] Sui G.-Y. Liu G.-C. Liu G.-Y. Gao Y.-Y. Deng Y. Wang W.-Y.. (2013). Is sunlight exposure a risk factor for age-related macular degeneration? A systematic review and meta-analysis. Br. J. Ophthalmol. 97, 389–394. doi: 10.1136/bjophthalmol-2012-302281, PMID: 23143904

[ref80] Sundelin S. Wihlmark U. Nilsson S. E. G. Brunk U. T. (1998). Lipofuscin accumulation in cultured retinal pigment epithelial cells reduces their phagocytic capacity. Curr. Eye Res. 17, 851–857. doi: 10.1080/02713689808951268, PMID: 9724002

[ref81] Taylor H. R. Muñoz B. West S. Bressler N. M. Bressler S. B. Rosenthal F. S. (1990). Visible light and risk of age-related macular degeneration. Trans. Am. Ophthalmol. Soc. 88, 163–173. 2095019 PMC1298584

[ref82] Theruveethi N. Bui B. V. Joshi M. B. Valiathan M. Ganeshrao S. B. Gopalakrishnan S.. (2022). Blue light-induced retinal neuronal injury and amelioration by commercially available blue light-blocking lenses. Life 12:243. doi: 10.3390/life12020243, PMID: 35207530 PMC8877890

[ref83] Thomson L. R. Toyoda Y. Langner A. Delori F. C. Garnett K. M. Craft N.. (2002). Elevated retinal zeaxanthin and prevention of light-induced photoreceptor cell death in quail. Invest. Ophthalmol. Vis. Sci. 43, 3538–3549, PMID: 12407166

[ref84] Tomany S. C. (2004). Sunlight and the 10-year incidence of age-related maculopathy: the beaver dam eye study. Arch. Ophthalmol. 122, 750–757. doi: 10.1001/archopht.122.5.750, PMID: 15136324

[ref85] Tomita Y. Shao Z. Cakir B. Kotoda Y. Fu Z. Smith L. E. H. (2020). An ex vivo choroid sprouting assay of ocular microvascular angiogenesis. J. Vis. Exp. 162:61677. doi: 10.3791/61677, PMID: 32831307 PMC8655367

[ref86] Van Der Burght B. W. Hansen M. Olsen J. Zhou J. Wu Y. Nissen M. H.. (2013). Early changes in gene expression induced by blue light irradiation of A2E-laden retinal pigment epithelial cells. Acta Ophthalmol. 91, e537–e545. doi: 10.1111/aos.12146, PMID: 23742627 PMC4955808

[ref87] Van Norren D. Gorgels T. G. M. F. (2011). The action Spectrum of photochemical damage to the retina: a review of monochromatic threshold data. Photochem. Photobiol. 87, 747–753. doi: 10.1111/j.1751-1097.2011.00921.x, PMID: 21410704

[ref88] Vila N. Siblini A. Esposito E. Bravo-Filho V. Zoroquiain P. Aldrees S.. (2017). Blue-light filtering alters angiogenic signaling in human retinal pigmented epithelial cells culture model. BMC Ophthalmol. 17:198. doi: 10.1186/s12886-017-0592-2, PMID: 29096624 PMC5667496

[ref89] Wang L. Yu X. Zhang D. Wen Y. Zhang L. Xia Y.. (2023). Long-term blue light exposure impairs mitochondrial dynamics in the retina in light-induced retinal degeneration in vivo and in vitro. J. Photochem. Photobiol. B 240:112654. doi: 10.1016/j.jphotobiol.2023.112654, PMID: 36724628

[ref90] Westlund B. S. Cai B. Zhou J. Sparrow J. R. (2009). Involvement of c-Abl, p53 and the MAP kinase JNK in the cell death program initiated in A2E-laden ARPE-19 cells by exposure to blue light. Apoptosis 14, 31–41. doi: 10.1007/s10495-008-0285-7, PMID: 19052872 PMC3099593

[ref91] Wong N. A. Bahmani H. (2022). A review of the current state of research on artificial blue light safety as it applies to digital devices. Heliyon 8:e10282. doi: 10.1016/j.heliyon.2022.e10282, PMID: 36042717 PMC9420367

[ref92] Wrona M. Korytowski W. Różanowska M. Sarna T. Truscott T. G. (2003). Cooperation of antioxidants in protection against photosensitized oxidation. Free Radic. Biol. Med. 35, 1319–1329. doi: 10.1016/j.freeradbiomed.2003.07.005, PMID: 14607531

[ref93] Wrona M. Różanowska M. Sarna T. (2004). Zeaxanthin in combination with ascorbic acid or α-tocopherol protects ARPE-19 cells against photosensitized peroxidation of lipids. Free Radic. Biol. Med. 36, 1094–1101. doi: 10.1016/j.freeradbiomed.2004.02.005 15082063

[ref94] Xie T. Cai J. Yao Y. Sun C. Yang Q. Wu M.. (2021). LXA4 protects against blue-light induced retinal degeneration in human A2E-laden RPE cells and Balb-c mice. Ann. Transl. Med. 9:1249. doi: 10.21037/atm-21-3390, PMID: 34532386 PMC8421929

[ref95] Xu Y. Li D. Su G. Cai S. (2022). The effect of A2E on lysosome membrane permeability during blue light-induced human RPEs apoptosis. BMC Ophthalmol. 22:241. doi: 10.1186/s12886-022-02464-1, PMID: 35641967 PMC9158258

[ref96] Xu Z. Zhang H. Zhang C. Yu S. Yuan J. Jin K.. (2023). REG1A protects retinal photoreceptors from blue light damage. Ann. N. Y. Acad. Sci. 1527, 60–74. doi: 10.1111/nyas.15045, PMID: 37531162

[ref97] Youssef M. A. Shehata A. R. Adly A. M. Ahmed M. R. Abo-Bakr H. F. Fawzy R. M.. (2024). Efficacy of repeated low-level red light (RLRL) therapy on myopia outcomes in children: a systematic review and meta-analysis. BMC Ophthalmol. 24:78. doi: 10.1186/s12886-024-03337-5, PMID: 38378527 PMC10877869

[ref98] Zhang J. (2005). Blue-light-induced apoptosis of cultured human retinal pigment epithelial cells *in vitro*. Chin. J. Ocul. Fundus Dis. SPIE (The International Society for Optics and Photonics) Publisher for Pautler 1990: U.S. Department of Defense (DoD) through the Defense Technical Information Center (DTIC). Available at: https://api.semanticscholar.org/CorpusID:87873832

[ref99] Zhang J. Sabarinathan R. Bubel T. Jia W. Williams D. R. Hunter J. J. (2024). Spectral dependence of light exposure on retinal pigment epithelium disruption in living primate retina. Investig. Opthalmology Vis. Sci. 65:43. doi: 10.1167/iovs.65.2.43, PMID: 38416456 PMC10910637

[ref100] Zhou H. Zhang H. Yu A. Xie J. (2018). Association between sunlight exposure and risk of age-related macular degeneration: a meta-analysis. BMC Ophthalmol. 18:331. doi: 10.1186/s12886-018-1004-y, PMID: 30572865 PMC6302450

